# *Campylobacter* Infection in Children in Malawi Is Common and Is Frequently Associated with Enteric Virus Co-Infections

**DOI:** 10.1371/journal.pone.0059663

**Published:** 2013-03-26

**Authors:** Jenifer Mason, Miren Iturriza-Gomara, Sarah J. O’Brien, Bagrey M. Ngwira, Winifred Dove, Martin C. J. Maiden, Nigel A. Cunliffe

**Affiliations:** 1 Institute of Infection and Global Health, University of Liverpool, Liverpool, United Kingdom; 2 Department of Community Health, College of Medicine, University of Malawi, Blantyre, Malawi; 3 Department of Zoology, University of Oxford, Oxford, United Kingdom; University of Aberdeen, United Kingdom

## Abstract

**Background:**

*Campylobacter* species are the most common cause of bacterial gastroenteritis in the developed world. However, comparatively few studies have determined the epidemiological features of campylobacteriosis in resource-poor settings.

**Methods:**

A total of 1,941 faecal specimens collected from symptomatic (diarrhoeic) children and 507 specimens from asymptomatic (non-diarrhoeic) children hospitalised in Blantyre, Malawi, between 1997 and 2007, and previously tested for the presence of rotavirus and norovirus, was analysed for *C. jejuni* and *C. coli* using a real time PCR assay.

**Results:**

*Campylobacter* species were detected in 415/1,941 (21%) of diarrhoeic children, with *C. jejuni* accounting for 85% of all cases. The median age of children with *Campylobacter* infection was 11 months (range 0.1–55 months), and was significantly higher than that for children with rotavirus and norovirus (6 months and 7 months respectively; P<0.001). Co-infection with either rotavirus or norovirus was noted in 41% of all cases in the diarrhoeic group. In contrast, the detection rate of *Campylobacter* in the non-diarrhoeic group was 14%, with viral co-infection identified in 16% of children with *Campylobacter*. There was no association between *Campylobacter* detection rate and season over the 10 year period.

**Discussion:**

Using molecular detection methodology in hospitalised Malawian children, we have demonstrated a high prevalence of *Campylobacter* infection, with frequent viral co-infection. The burden of *Campylobacter* infection in young African children may be greater than previously recognised.

## Introduction

It is estimated that 3.552 million children under the age of five years die each year in Africa; diarrhoeal disease accounts for 11% of these deaths [1]. Diarrhoea also contributes to childhood morbidity, particularly malnutrition and growth stunting [2], [3]. The introduction of intervention strategies, such as provision of oral rehydration therapy, has had a positive impact on reducing childhood diarrhoea mortality, with an estimated annual reduction of diarrhoeal deaths in Africa since 2000 of 3.7% [1]; however diarrhoea remains a leading cause of child mortality and morbidity in this region. The importance of pathogens such as rotavirus and enterotoxigenic *Escherichia coli* in the aetiology of severe childhood diarrhoea in developing countries is well recognised [4], [5]. However the role of *Campylobacter* is less well understood.

*Campylobacter* is a fastidious gram negative bacterium and *C. jejuni* and *C. coli* are considered the most common cause of bacterial gastroenteritis in the developed world. The clinical presentation ranges from mild watery to severe inflammatory diarrhoea which may be complicated by post infectious sequelae such as Guillain-Barré Syndrome [6]. Using bacterial culture methodology, estimates of the prevalence of *Campylobacter* infection in young children with diarrhoea in Sub Saharan Africa range from 1.5% to 18% [7], [8], [9], [10], [11]. While molecular techniques have been developed and employed for *Campylobacter* detection in epidemiological studies in developed countries, such methods have not been widely adopted in Sub Saharan Africa. Where molecular detection (notably PCR) was used to examine for *Campylobacter* species in adults and children with diarrhoea in South Africa the prevalence estimates of *C. jejuni, C. coli* and *C. concisus* were 12%, 7.5% and 2.7% respectively; however only 34 of the 255 samples analysed were from children <5 years of age [12].

As part of a long-term research programme investigating viral gastroenteritis in children in Malawi, we have collected stool samples since 1997 from children <5 years of age admitted to the Queen Elizabeth Central Hospital (QECH), Blantyre, Malawi with moderate to severe diarrhoea [5]. We have now examined stored faecal specimens using a real time PCR assay to determine the prevalence and epidemiological features of *Campylobacter* infection in this population.

## Methods

### Ethics Statement

Written, informed consent was obtained from the child’s parent or guardian prior to enrolment. Ethical approval was obtained from the Malawi National Health Sciences Research Committee.

### Study Site

The QECH is a large government run tertiary referral hospital in the southern Malawian city of Blantyre, which has a population of approximately 1 million living in urban and peri-urban settlements.

### Specimen Collection

Enrolment and data collection procedures have previously been described in detail [5]. Briefly, children age <5 years admitted to the QECH with ≥3 loose or watery stools within a 24 hour period for <14 days, were eligible for inclusion. Children were enrolled Monday to Friday, 9 am to 5 pm, from 1^st^ July 1997 to 30^th^ June 2007. A second group of children <5 years of age without diarrhoea, who were admitted to the QECH with conditions such as malaria and respiratory infections, were enrolled between 1997 and 1999. A single faecal specimen was obtained from each child. Clinical data (illness severity, blood in stools etc.) were not routinely gathered. Following EIA testing for rotavirus [5], remaining samples were shipped to the University of Liverpool and stored at −80°C until testing for norovirus by real time PCR [13] and for *Campylobacter* (this study).

### *Campylobacter* Testing

Of 2,458 faecal specimens collected from hospitalised diarrhoeic children in the primary study [5], 1,941 were available for testing for *Campylobacter* infection, together with 507 samples from children admitted to hospital without diarrhoea. DNA was extracted from all samples using an automated extractor (Qiasymphony, Qiagen). The presence of a 95 base pair fragment of the *mapA* gene of *C. jejuni* and a 103 base pair fragment of the *ceuE* gene of *C. coli* were detected using a real time PCR method [14].

### Statistical Analysis

Data were analysed using “IBM SPSS Statistics Data Editor” version 11. Categorical data were analysed using *Chi^2^* test and continuous data using paired T-tests. A *p*-value of <0.05 was considered significant.

## Results

### Characteristics of the Study Population

In total 2,448 samples was analysed; 1,941 from diarrhoeic children and 507 from non-diarrhoeic children. The median age of children in the diarrhoeic group was 9 months (range 0–55 months) and in the non-diarrhoeic group was 6 months (range 1–50 months). The diarrhoeic group contained 55% males and the non-diarrhoeic group 52% males.

### Prevalence

Over the 10 year study period (1997–2007) *Campylobacter* was detected in 415/1941 (21%) of diarrhoeic specimens. For the two year period (1997–1999) in which faecal specimens from non-diarrhoeic children were collected, the detection rate of *Campylobacter* was significantly higher in the diarrhoeic specimens than the non-diarrhoeic specimens (28% vs. 14%; *p*<0.001) (Table 1). Of the *Campylobacter* species detected between 1997 and 1999, *C. coli* comprised 10% and 4% of all *Campylobacter* from diarrhoeic and non-diarrhoeic specimens respectively (*p<*0.001). There was no statistical difference in the *Campylobacter* cycle threshold values obtained from diarrhoeic vs. non-diarrhoeic specimens (data not shown).

**Table 1:** Age Distribution of *Campylobacter* infection.

	Age Group	Total	*Campylobacter* positive	*Campylobacter*+virus	*Campylobacter* alone
Diarrhoeic (1997–2007)	0–2 months	156	31 (20%)	20 (13%)	11 (7%)
	3–5 months	412	83 (20%)	43 (10%)	40 (10%)
	6–11 months	673	153 (23%)	70 (10%)	83 (12%)
	12–17 months	418	82 (20%)	28 (7%)	54 (13%)
	>18 months	282	66 (23%)	7 (2%)	59 (21%)
	Total : 1997–1999	738	206 (28%)	87 (12%)	119 (16%)
	Total: 1997–2007	1941	415 (21%)	168 (9%)	247 (13%)
Non-diarrhoeic (1997–1999)	0–2 months	140	7 (5%)	0	7 (5%)
	3–5 months	104	14 (13%)	0	14 (13%)
	6–11 months	81	15 (19%)	6 (7%)	9 (11%)
	12–17 months	80	12 (15%)	2 (3%)	10 (13%)
	>18 months	102	21(21%)	3 (3%)	18 (18%)
	Total: 1997–1999	507	69 (14%)	11 (2%)	58 (11%)
**χ^2^** (Diarrhoeic vs. non-diarrhoeic 1997–1999)			35.7	38.3	5.4
***p*** ** value**			<0.001	<0.001	0.02

### Age Distribution

**Figure 1:**
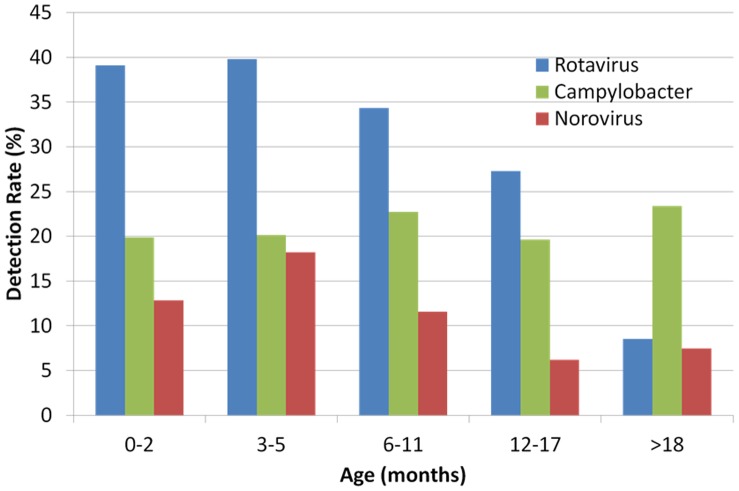
Age distribution of *Campylobacter*, rotavirus and norovirus infections in Malawian children with acute diarrhoea, 1997–2007.

The median age of children with diarrhoea in whom *Campylobacter* was detected was 11 months (range 0.1–55 months) which was higher than the age of children with rotavirus or norovirus (median age 6 months and 7 months respectively (*p*<0.001)). The detection rate of *Campylobacter* was relatively constant across all age groups, in contrast to the detection rate of rotavirus and norovirus which decreased with age (Figure 1). The median age of non-diarrhoeic children with *Campylobacter* infection was 11 months (range 1–34 months); the detection rate increased from 5% of children in the 0–2 month age group to 20% of children age >18 months (Table 1).

### Viral Co-infections

In the diarrhoeic group 40% of children with *Campylobacter* had an enteric virus co-infection. These co-infections occurred predominantly in children <1 year of age with 50% of all *Campylobacter* cases in this age group also having either rotavirus or norovirus in the specimen. In the non-diarrhoeic group 16% of children with *Campylobacter* in the specimen also had a viral co-infection (Table 1). Although the overall prevalence of *Campylobacter* was significantly higher in the diarrhoeic than in the non-diarrhoeic group, when single infections were considered (i.e. in the absence of either rotavirus or norovirus) this difference is less pronounced (*Campylobacter* detection rate of 16% in the diarrhoeic vs. 11% in the non-diarrhoeic group, *p = *0.02).

### Seasonality

The detection rate of *Campylobacter* did not vary consistently by month or season of specimen collection during the 10 year study period. In total 49% of *Campylobacter* positive specimens in the diarrhoeic group occurred in the dry season (May to October) and 51% in the wet season (November to April). In the non-diarrhoeic group 41% and 59% of positive specimens occurred in the dry and wet season respectively (*p = *0.89; data not shown).

## Discussion

In this large study of *Campylobacter* infection in Malawian children, using a sensitive molecular assay we documented *Campylobacter* in 21% of specimens obtained from children <5 years of age hospitalised with diarrhoea. There are no previous data describing the prevalence of *Campylobacter* infection in Malawi. Estimates of the prevalence of *Campylobacter* infection in hospitalised children <5 years of age with diarrhoea in Sub Saharan Africa range from 1.5% in Botswana [12], 1.7% in Mozambique [8], 9% in Uganda [7], 11% in The Central African Republic [9] to 18% in Tanzania [10]. In community settings the detection rate of *Campylobacter* in diarrhoeal faecal specimens among children <5 years of age has been estimated at 15.9% in The Central African Republic [15], 3.3% in Djibouti [16] and 0.8% in Guinea-Bissau [17]. This variation in detection rate across Sub Saharan Africa may reflect the technical difficulties of isolating *Campylobacter* species in resource poor settings because of its fastidious growth requirements and/or the relative insensitivity of some culture techniques.

Comparing culture and PCR in the detection of *C. jejuni* and *C*. *coli* in diarrhoeal faecal specimens in Ireland suggested that culture alone detected only 55% of all cases [18]. In the UK, *Campylobacter* detection rates in community diarrhoea cases increased from 4.0% by direct culture, to 5.0% after a faecal enrichment procedure was added, to 15% by PCR [19]. A study in South Africa of 255 adults and children with diarrhoea and 67 children without diarrhoea, demonstrated similar overall rates of *Campylobacter* using PCR to those reported in the current Malawi study (19.6% in symptomatic patients and 11% in asymptomatic patients) [12]; however the prevalence in children <5 years of age with diarrhoea was 11% compared with 21% in the current study. Thus our data suggest that the detection rate of *C. jejuni* and *C. coli* using a sensitive and specific PCR assay in faecal specimens of children <5 years of age hospitalised with diarrhoea in Sub Saharan Africa may be higher than previously reported.

Of note, we documented a relative over-representation of *C. coli* compared with *C. jejuni* in the faecal specimens of diarrhoeic compared with non-diarrhoeic children. A similar pattern was reported in Tanzania where *C. coli* was present in 20% of diarrhoeic and 6% of non-diarrhoeic specimens from adults and children [10]. In the developed world *C. coli* is particularly associated with chicken [20] and the over-representation of *C. coli* in children with diarrhoea raises the possibility that poultry may be a particularly important source of infection in this group. *Campylobacter spp*. other than *C. jejuni* and *C. coli*, which require either non-selective culture or specific molecular techniques for their optimal detection, are reported to be common in some studies. For example in South Africa *C. concisus* and *C. upsaliensis* accounted for 23%, and *C. coli* for 3%, of all *Campylobacter* and related species isolated [21] and in Ireland using PCR *C. ureolyticus* accounted for 22.3% of all *Campylobacter* species detected [22]. Although both *C. jejuni* and *C. coli* were commonly detected among hospitalised Malawian children with diarrhoea, the contribution of other *Campylobacter* species has not yet been determined.

The prevalence of *Campylobacter* remained relatively constant in children up to 5 years of age in the current study suggesting that unlike viral infections, *Campylobacter* may make a relatively greater contribution to diarrhoeal disease in children aged >12 months. It is possible that repeated exposure to *Campylobacter* species from environmental sources throughout childhood may explain the relatively high prevalence in children >1 year of age; however further clinical, epidemiological, and immunological studies are required to confirm the sources of transmission, environmental reservoirs and the role of *Campylobacter* infection in diarrhoeal disease in this setting. Where detailed age related prevalence rates have been determined in children in Sub Saharan Africa similar patterns have been observed, for example a large cohort study in The Central African Republic reported an average *Campylobacter* detection rate of 9.5% across all age groups [9]. Although deaths in children secondary to diarrhoea are decreasing worldwide, diarrhoea morbidity remains constant, particularly in older children [23]. Given the association of childhood diarrhoea with growth stunting in this population [24] the contribution of *Campylobacter* to the burden of diarrhoeal disease children >1 year of age requires further elucidation.

A significant proportion of diarrhoeal specimens in which *Campylobacter* was detected also contained norovirus or rotavirus, particularly in children <12 months of age. Given the high prevalence of *Campylobacter* in non-diarrhoeic specimens, its detection in diarrhoeic specimens may represent “flushing out” of organisms in diarrhoeal episodes caused by a viral pathogen. We do not have data on the presence of other bacterial or parasitic organisms. We are therefore unable to ascertain which, if any, pathogen is the causative agent of the diarrhoeal episode, although we speculate that *Campylobacter* when detected alone in older children may be more likely to be the causative agent of disease. Furthermore, although there was a significant difference in the rate of detection of *Campylobacter* in isolation between the diarrhoeic and non-diarrhoeic groups (16% vs. 11% respectively), further case control studies are required to confirm this finding. Mixed infections involving *Campylobacter* have been described previously; one large (n = 3,038) surveillance study of diarrhoea in hospitalised children <5 years of age in Bangladesh reported that 59% of all *Campylobacter* infections were associated with at least one other bacterial or protozoan pathogen [25]. Few studies have reported mixed infections of *Campylobacter* and viral pathogens; a cohort study in India demonstrated that rotavirus was associated with 11/18 *Campylobacter* infections in children <5 years of age [26]. Since data from in-vitro cell culture systems suggest that viral co-infection increases the adhesion and invasion of pathogenic *Campylobacter* species [27], further work should explore the clinical consequences of co-infection with viral enteric pathogens on the pathogenesis of *Campylobacter* infection.

*Campylobacter* was detected in 14% of non-diarrhoeic children who were recruited over a two-year period. Since no preceding clinical or microbiological data are available for these children, we are unable to determine whether this finding represents asymptomatic colonisation or extended excretion after resolution of a *Campylobacter* diarrhoeal episode. Asymptomatic colonisation appears to occur frequently in children in Sub Saharan Africa; one longitudinal study reported that 41.7% of all children in a community birth cohort were asymptomatically colonised with *Campylobacter* within the first 6 months of life [15]. This phenomenon does not appear to occur in the developed world with the exception of workers occupationally exposed to *Campylobacter* [28]. It is thought that regular exposure to *Campylobacter* within an abattoir or farm environment results in immune tolerance towards *Campylobacter* species and hence facilitates colonisation [29]. It is feasible that a similar response occurs in children in Sub Saharan Africa explaining the observed high rates of asymptomatic infection. Given the high sensitivity and specificity of the assay used in this study it is also possible that the detection rates seen in the control cohort could represent post excretion following a diarrhoeal episode as *Campylobacter* is known to be excreted for up to 12 weeks post infection [30].

This study highlights specific differences in the epidemiology of *Campylobacter* in developed compared to developing countries. Firstly, in the UK, the detection rate of *Campylobacter* in children with and without diarrhoea is less than that seen in children in Sub Saharan Africa (6.8% and 2% in diarrhoeal and non-diarrhoeal children in the UK respectively) [14]; secondly, there are age specific differences in detection rates in children in the UK with prevalence increasing throughout childhood [14]; and lastly there is a strong seasonal association in temperate climates with a peak in incidence occurring during the warmer months whereas no seasonal association was seen in Malawi [31]. These differences may be explained by a number of factors including differences in the routes of transmission between the two settings; in the developed world the majority of *Campylobacter* strains causing human infections can be epidemiologically linked to strains that colonise poultry, with the main route of transmission of the pathogen thought to be through the handling and consumption of contaminated meat [32]. Although there are no comparative epidemiological studies linking poultry and human strains in Sub Saharan Africa, up to 40% of commercial chickens in Senegal and 60% in South Africa are colonised with *Campylobacter* [33], [34]. Furthermore, strains colonising chickens in Senegal are genetically similar to strains colonising chickens in the UK, The Netherlands and United States suggesting that the host association of *Campylobacter* genotypes transcends geographical boundaries [35]. Chicken is a widely consumed meat in Malawi and many families keep chicken flocks which are routinely housed within the human dwelling including in food preparation areas [36]. Thus in contrast to the developed world, there is increased potential for acquisition and spread of zoonotic and foodborne disease such as *Campylobacter* which may account for the higher prevalence rates reported in this and other studies.

In conclusion, this large study of children hospitalised with diarrhoea in Malawi suggests that the burden of *Campylobacter* is higher than previously appreciated, and is frequently identified in association with concomitant rotavirus and/or norovirus infection. Given the recent introduction of rotavirus vaccines into childhood immunisation programmes in Malawi and other parts of Sub Saharan Africa it is predicted that bacterial pathogens including *Campylobacter* could play a more prominent role in the aetiology of diarrhoeal disease in young children in this region in the future. An improved understanding of the clinical features, epidemiology, and pathogenesis of *Campylobacter* infection in Sub Saharan Africa will inform future prevention strategies against this foodborne zoonotic pathogen.
